# Measuring normal ocular torsion and its variation by fundus photography in children between 5-15 years of age

**DOI:** 10.4103/0301-4738.67060

**Published:** 2010

**Authors:** Jitendra Jethani, G Seethapathy, Jaypraksh Purohit, Deepak Shah

**Affiliations:** Dr. Thakorbhai V Patel Eye Institute, Salatwada, Baroda- 390 001, India; 1 Manipal Hospitals, Salem, India

**Keywords:** Children, fundus photography, ocular torsion

## Abstract

Cycloposition has been measured by various methods; however, fundus photography is the most reliable method to evaluate the torsion objectively. We did a prospective study to find out the disc foveal angle (DFA) and its variation in children without squint. We included 210 eyes of 105 children between the ages of 5-15 years. DFA was calculated using standard technique after taking a fundus photograph. The cycloplegic refraction was done and compared. The mean age was 10.6 ± 2.5 years. Mean DFA in right eye (RE) was 6.49 ± 3.25° (0-13°) and in left eye (LE) was 5.80 ± 3.29° (0-12°). The difference between the RE and LE was statistically not significant (*P*=0.131) (mean 1.15 ±1.39°). Mean DFA in emmetropic children was 6.1° ± 3.4° (n=112 eyes). DFA varies widely in children. The difference observed in DFA measurement for eyes with various refractive errors were compared with DFA measurements for emmetropic eyes.

Cycloposition (extent of ocular torsion) has been measured by various subjective methods including perimetry, double maddox rod test, Bagolini’s glasses, indirect ophthalmoscopy lens, slit-lamp biomicroscopy and synoptophore. A reliable method to evaluate ocular torsion objectively is fundus photography.[1–5] However, wide variations have been reported in the measured results of the disc foveal angle (DFA) which is formed at the optic disc center between the horizontal meridian and the line joining the center of disc and foveal center.[2,3] DFA is indicative of the cycloposition of the eyes. The aim of the study was to evaluate the DFA and to find its correlation with possible influencing factors in children between 5–15 years of age.

## Materials and Methods

**Figure 1:**
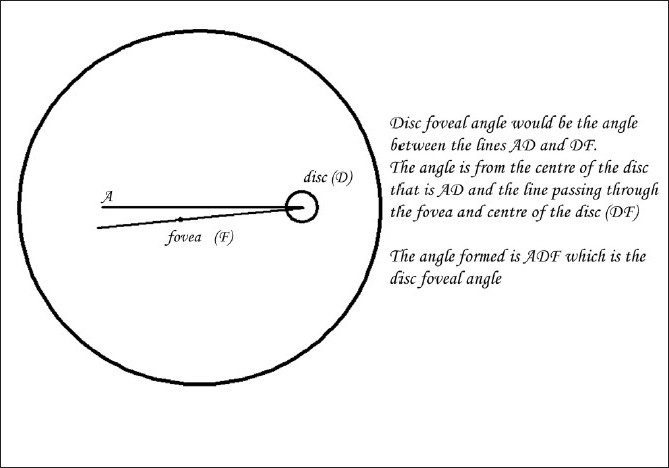
Schematic diagram shows the disc foveal angle (DFA)

A total of 105 children (210 eyes) were included in the study. The patients (and /parents) were briefed about the procedure and appropriate consent obtained for fundus photography. The age, sex and refractive errors of all patients were noted. We excluded all the patients with hazy media, abnormal muscle balance, retinal pathology and any manifest deviation. Photographic documentation of the ocular fundi of our patients was done by one of the authors (JP) using a TRC- 50DX (Topcon, Japan) fundus camera taking care that the subject’s head was well aligned - the side marks and chin rest were taken as a guide. Wide-field (50°) fundus photographs were taken. Children who were not cooperative or if the authors found were not able to keep their head straight were excluded from the study. Photographs were taken through the dilated pupil after instilling tropicamide 1% eye drops. The DFA was calculated from a well-focused single still photograph using IMAGEnet software (Topcon, Japan) and a protractor. To obtain the measurement of DFA, two lines were drawn; one straight line (horizontal meridian) passing through the center of the disc [Fig. 1] (AD) and another line passing through the point D (center of the disc) and the Fovea (F) (DF). The angle between the fovea and the geometric centre of the disc (between the lines AD and DF) was measured in order to obtain the DFA (< ADF) [Fig. 1].

All the measurements were performed by one of the authors (JJ). The readings were recorded on an MS Excel spreadsheet and the data analyzed and compared. The ‘*P*’ value was calculated by Welch two-sample t-test and a ‘*P*’ value < 0.05 was considered significant.

## Results

A total of 105 patients (210 eyes) were included in the study. Mean age was 10.6 ± 2.5 years (5-15 years). We analyzed the values of DFA under two perspectives. Firstly, the sample was stratified into refractive subcategories namely emmetropes, myopes, hyperopes, simple myopic astigmatism (SMA), compound myopic astigmatism (CMA), simple hyperopic astigmatism (SHA) and compound hyperopic astigmatism (CHA) and mixed astigmatism (MA) patients. The *P* value was calculated by Welch two-sample t-test of the mean values with the emmetropic children [Table 1]. Secondly, we tabulated the distribution of ocular torsion in emmetropic eyes and its correlation with age as shown in Table 2.

**Table 1:** Comparison of mean and standard deviation of disc foveal angle in various refractive errors and in emmetropia. Also the *P* value of comparison of these values with emmetropia

Parameters	Mean (degrees)	SD	Sample size (n=210)	*P* value with emmetrope
Emmetrope	6.1	3.4	112	Invalid
Myope	6.1	3.3	20	0.981
Hyperope	5.6	3.7	15	0.641
MA	4.7	3.6	6	0.382
CMA	7.1	2.9	27	0.108
CHA	3.7	0.6	3	0.001
SMA	6.1	3.3	15	0.955
SHA	6.7	3.5	12	0.587

MA: Mixed astigmatism, CMA: Compound myopic astigmatism, CHA: Compound hypermetropic astigmatism, SMA: Simple myopic astigmatism, SHA: Simple hypermetropic astigmatism

**Table 2:** The distribution of torsion in emmetropic eyes in various age groups

Age in years	Mean ± SD (DFA) in degrees	Total number of eyes (n=120)
5	6 ± 4.17	8
6	6.83 ± 1.17	5
7	3.16 ± 4.21	6
8	7.60 ± 2.32	10
9	5.61 ± 3.37	13
10	6.12 ± 3.99	15
11	6.28 ± 3.77	7
12	6.45 ± 3.51	21
13	7.0 ± 2.12	14
14	6.8 ± 2.94	5
15	4.5 ± 3.77	8

SD: Standard deviation

## Discussion

The DFA measured objectively by fundus photography has wide variations.[4] The optic nerve head to fovea distance differed more vertically than horizontally both inter-individually and intra-individually.[6] This distance, however, does not allow for meaningful determination of the location of the fovea in eyes where morphologic changes have occurred since the angle of rotation of the fovea in relation to the optic nerve head is relatively stable.

de Ancos *et al*.[5] with fundus photography established a mean DFA of 7.030 ± 2.90. Williams and Wilkinson[7] found the foveal center to be, on average, 6.110 ± 3.30 below a horizontal line bisecting the nerve head in 446 normal adult eyes with fundus photography. Kothari *et al*.[4] in 36 eyes found an average of 6.10 ± 4.30 with fundus photographic technique. Bixenman *et al*.[8] found that the average location of fovea was around 0.3 disc diameters below a horizontal line extended through the geometric center of the optic disc. They reported a DFA of 7.20 ± 2.50 in 100 eyes (50 subjects). Lefevre *et al*.[3] have also studied the DFA by retinal photography. All their patients had normal oculomotor function and were close to emmetropia. The DFA followed a Gaussian distribution with a mean of 6.30 ± 3.40. In our study, the DFA varies between 3.160 and 7.600 When tabulated for emmetropes between 5 and 15 years of age.

The individual right-left asymmetries of less than 40 by fundus photography and less than 70 by monocular perimetry are considered normal.[1] Kothari *et al*.[4] in their study found a mean inter-ocular difference of 5.50 ± 4.60 using fundus photographic technique to measure the DFA. However, the patients included had a variety of refractive errors (no details are given).[4] Besides, Bixenman *et al*.[8] had previously reported a mean inter-eye difference of 1.60 ± 1.20. Variation between the right and left eyes of the same individual was not statistically significant in our study in the emmetropic population (mean 1.150 ± 1.390). Keilhaver *et al*.[9] in their study suggested that ametropia arising from axial length differences can lead to different angular distances between the macula and optic disc due to magnification. However, Roschneider *et al*. have not noted any significant change in the angular distance (DFA) with age or with any degree of myopia.[6]

In our study, we found that both age and type of refractive error do not have any statistically significant association with the DFA. The variation in DFA could be due to biological variability.

## Conclusion

We have established the physiological range of DFA in children in the Indian population. The minimal intraindividual variation (inter-eye variation) in children (not cooperative for the subjective tests) may help regarding their torsional status. Since the inter-eye variability is small, if the inter-eye DFA variability is large in a child, the child could have a torsional disturbance and should be evaluated for the same.
